# Circulating endothelial progenitor cells and endothelial cells in moyamoya disease

**DOI:** 10.1002/brb3.1035

**Published:** 2018-08-23

**Authors:** Xiang‐Yang Bao, Yan‐Na Fan, Yi Liu, Qian‐Nan Wang, Yong Zhang, Bing Zhu, Bing Liu, Lian Duan

**Affiliations:** ^1^ Department of Neurosurgery The Center for Cerebral Vascular Disease PLA 307th Hospital, PLA Beijing China; ^2^ Center of Interventional Radiology for Oncology 307th Hospital, PLA Beijing China; ^3^ Lab of Tumor Molecular 307th Hospital, PLA Beijing China

**Keywords:** circulating endothelial cells, endothelial progenitor cells, flow cytometry, moyamoya disease

## Abstract

**Introduction:**

There is no well‐recognized biomarker for accurately predicting outcome in the presence of moyamoya disease (MMD), a progressive occlusive cerebrovascular disease of the internal carotid arteries or their branches. The aim of this study was to investigate the presence of endothelial progenitor cells (EPCs) and circulating endothelial cells (CECs) in MMD and correlate the findings with clinical features.

**Methods:**

Patients with MMD (*n* = 66) were compared with healthy controls (*n* = 81). Blood samples were obtained from an antecubital vein and analyzed using flow cytometry. EPCs were defined as CD31^+^ CD45^dim^ CD34^br^ CD133^+^ and CECs as CD31^br^ CD45^−^ CD34^dim^ CD133^−^. Univariate and multivariate linear regression analyses were carried out.

**Results:**

The CEC counts were significantly higher in the patients than in the controls (*p* = 0.008). In multivariate analysis, EPC counts were independently associated with age of patients with MMD (*p* = 0.049) and CEC counts were independently negatively associated with concomitant disease such as hypertension, diabetes mellitus, and coronary heart disease (*p* = 0.034).

**Conclusions:**

This is the first study to investigate the presence of CECs in the plasma of patients with MMD, and the amount of CECs was negatively correlated with concomitant disease in these patients.

## 1 INTRODUCTION

Moyamoya disease (MMD) is a progressive occlusive cerebrovascular disease of the internal carotid arteries or their branches with compensatory development of a fine collateral vascular network at the base of the brain (moyamoya vessels) (Suzuki & Takaku, 1969; Takeuchi & Shimizu, 1957). The most characteristic feature of MMD pathology is thickening of the intima with the proliferation of smooth muscle cells (Phi et al., 2017). The etiology of MMD remains unknown. Genetic factors may play a role in about 10% of patients (Kim et al., 2010). In East Asian countries, the *Ring Finger protein 213* (*RNF213*) gene is an important susceptibility gene for MMD (Bang, Chung, et al., 2016; Kim, 2016). Factors that might play a role in the pathophysiology include circulating endothelial progenitor cells (EPCs), circulating smooth muscle progenitor cells (SPCs), and cytokines related to vascular remodeling and angiogenesis (Bang, Fujimura, & Kim, 2016). Prior studies to predict the presence of MMD using various invasive or noninvasive approaches including inflammatory molecules, cytokines, chemokines, and growth factors in serum and cerebrospinal fluid (CSF) have revealed varying levels of discriminatory capacity (Bedini et al., 2016). There is no well‐recognized surrogate biomarker that can accurately predict the outcome of confirmed MMD, although there have been efforts to find serological biomarkers in MMD, such as serum levels of matrix metalloproteases, cytokines, trophic factors, and caveolin‐1. Surgical revascularization is used for preventing stroke in patients with MMD (Bang, Fujimura, et al., 2016; Kim, Oh, Bang, Kim, & Cho, 2016). The strategy for revascularization is to use the external carotid system to augment intracranial blood flow (Kim et al., 2016).

Endothelial progenitor cells (EPCs) represent some bone marrow‐derived cells described and isolated for the first time from human peripheral blood by Asahara et al. (1997). Following hypoxia or vascular injury, EPCs are recruited into the systemic circulation by secretion of various proangiogenic cytokines (Heil, Ziegelhoeffer, Mees, & Schaper, 2004; Khakoo & Finkel, 2005; Takahashi et al., 1999; Timmermans et al., 2009). They have the capacity to proliferate, migrate, and differentiate into endothelial‐like cells without acquiring the features of mature endothelial cell (EC) markers (Khakoo & Finkel, 2005). Patients with MMD have been found to have increased levels of EPCs (Rafat, Beck, Pena‐Tapia, Schmiedek, & Vajkoczy, 2009). However, there have been controversial results about the levels of EPCs in MMD. For example, Kim et al. (2010) reported that the level and function of EPCs in childhood MMD were decreased, and Jung et al. (2008) showed that the functional activity of EPCs was impaired in MMD. A subpopulation of EPCs in MMD has been identified as colony‐forming unit and outgrowth cells (Jung et al., 2008). As some EPCs are known to contribute to neovascularization by differentiating into mature ECs while other EPC subsets function via paracrine effects, circulating cells have been hypothesized to be involved in vascular remodeling in MMD. They stimulate angiogenic activity of resting endothelial cells leading to their proliferation and sprouting (Asahara et al., 1999). EPCs have been investigated to better understand and characterize MMD pathogenesis, but the results have been conflicting (Jung et al., 2008; Kim et al., 2010; Rafat et al., 2009; Yoshihara et al., 2008). Lack of uniformity in terminology and methodology has caused considerable confusion in characterizing EPCs, and this may be the predominant reason for the variability in published results. Several authors have used polychromatic flow cytometry to better characterize these circulating cells and have reported that the vast majority of EPCs are comprised of proangiogenic hematopoietic cells that do not display in vivo vessel‐forming ability, but promote recovery of vascular endothelial functions via paracrine mechanisms (Duda, Cohen, Scadden, & Jain, 2007; Khan, Solomon, & McCoy, 2005).

Circulating endothelial cells (CECs), first described in 1970 (Bouvier, Cintron, Bernhardt, & Spaet, 1970; Hladovec & Rossamann, 1973), have been recognized as markers of vascular injury. CECs can be derived from the vascular wall (mature CECs), or recruited from the bone marrow, as endothelial progenitor cells (EPCs) (Asahara et al., 1997), in response to various stimuli such as ischemia, vascular trauma, and acute myocardial infarction, sickle cell anemia, vasculitis, and pulmonary hypertension. Both EPCs and CECs have been defined on the basis of surface markers (Goon, Lip, Boos, Stonelake, & Blann, 2006). Several authors have reported increased numbers of CECs in response to a variety of stresses or pathological conditions (Koc, Bihorac, & Segal, 2003). The presence of these cells has also been associated with angiogenic potential (Khan et al., 2005). Although there have been a number of studies that focused on CECs, no study has investigated the presence of CECs in the plasma of patients with MMD in the clinical setting. Therefore, the aim of our study was to investigate the presence of EPCs and CECs in patients with MMD, and to correlate the findings with clinical features of the patients.

## 2 MATERIALS AND METHODS

### 2.1 Patient selection

We identified all consecutive adult patients with MMD (*n* = 66) at the Department of Neurosurgery, 307 Hospital PLA, Beijing, China, from November 2014 through May 2015. The diagnostic criterion for MMD is that cerebral angiography or magnetic resonance angiography must show at least the following findings: (1) stenosis or occlusion of the terminal portion of the intracranial internal carotid artery or proximal portions of the anterior and/or the middle cerebral artery; (2) abnormal moyamoya vessel networks in the vicinity of the occlusive or stenotic lesions in the arterial phase; and (3) bilaterality of findings (1) and (2) must be present (Duda et al., 2007). Sixteen patients presenting with unilateral MMD were included. All patients with MMD were nonhemorrhagic. The median sampling time after stroke onset was 4 months (range: 2.5 months to 6 years). All patients were studied before undergoing bypass surgery. Exclusion criteria were intracranial atherosclerosis, meningitis, Down syndrome, systemic vasculitis, acute stroke, hyperthyroidism, neurofibromatosis, leptospiral infection, or prior skull‐base radiation therapy. Most of these conditions had been excluded to accurately diagnose MMD (Bang, Fujimura, et al., 2016; Fujimura & Tominaga, 2015). The autoimmune disease hyperthyroidism was excluded because autoimmune diseases can cause MMD. For controls, we recruited 81 healthy volunteers aged 18 years or older from our department staff, hereafter referred to as healthy controls. They were matched mainly on the basis of age and sex. The volunteers were scanned with transcranial Doppler to exclude individuals with cerebrovascular disease. Patients in whom MMD was associated with endothelial injury, such as trauma, cancer, antiphospholipid antibodies, surgery within the last 3 months, pregnancy, renal disease, or hepatic disease were excluded from the study, and the same exclusion criteria were applied for the controls.

Clinical records, including hospital charts, clinic notes, serological examination, and radiological studies, were reviewed. History of hypertension, diabetes, and coronary heart disease was recorded. The angiographic stage was evaluated according to Suzuki's classification (Suzuki & Takaku, 1969); the higher Suzuki grade was used when measures differed from side to side. Based upon the morphology of the moyamoya vessels, all patients were divided into three groups: no moyamoya vessels (Suzuki stages 1 and 6), a small amount of moyamoya vessels (Suzuki stages 2 and 5), and a large amount of moyamoya vessels (Suzuki stages 3 and 4). The study was approved by the Research Ethics Board at 307 Hospital, and all the individuals signed a written informed consent. All the procedures utilized in this study were in agreement with the Declaration of Helsinki.

### 2.2 Blood sampling and flow cytometry analyses

To evaluate CECs and EPCs, blood samples were drawn from an antecubital vein. Venous blood was collected in 5‐ml acid–citrate–dextrose tubes and processed within 3 hr of collection. Blood samples were kept at 4°C throughout the procedure. Whole‐peripheral blood samples were analyzed by flow cytometry (Duda et al., 2007). During the procedure, fresh samples were centrifuged at 700 *g* for 20 min with no brake. The upper phase (plasma) was gently removed into a separate tube and stored in 0.25‐ml aliquots. The lower phase containing the blood cells was resuspended using 10 ml of cold 1 × PBS containing 0.5% (w/v) BSA and 1.5 mM EDTA and centrifuged at 700 *g* for 20 min with no brake for a second time. The upper phase was removed and discarded; the cell pellet was resuspended, and 2.5 ml was transferred into a separate tube and kept on ice. Concomitantly, 500 μl of samples was also transferred into one isotype control and four sample tubes and the appropriate antibodies (CD31‐FITC, CD34‐APC, CD45‐PerCP, and CD133‐PE) were added. Then, 9 ml of ACK lysing buffer was added (to lyse red blood cells), vortexed briefly, and incubated at room temperature (18–25°C) for 3 min. The cell suspension was washed twice with 9 ml of cold regular 1 × PBS and centrifuged at 250 *g* at 4°C with brake for 5 min. The cell pellet was resuspended in 500 μl of 1 × PBS, and the samples were filtered through a 40‐μm cell strainer into 5‐ml BD Falcon tubes. The tubes were held at 4°C (or on ice) in the dark before acquisition on the flow cytometer. Flow cytometry was performed in the Flow Cytometry Core Laboratory with a BD FACSCanto II (Becton–Dickinson) flow cytometer. An acquisition gate was established that included mononuclear cells (PBMCs) but excluded most granulocytes and debris; 10^6^ mononuclear events were routinely collected to determine this population. Finally, Boolean analysis using a combination of specific surface markers was applied. EPCs were defined as CD31^+^CD45^dim^CD34^br^CD133^+^, and CECs were identified as CD31^br^CD45^−^CD34^dim^CD133^−^. All analyses were performed using FlowJo (version 7.6 for MacIntosh; Treestar Inc.), and both EPC and CEC levels are reported as a concentration as well as a percentage of PBMCs.

### 2.3 Statistical analysis

All the clinical characteristic data are presented as means ± standard deviations (*SD*) with a range (min. to max.) for continuous variables and *n* (%) for categorical ones. The dispersion in age between patients with MMD and healthy controls was presented as mean ± *SD* and compared using two‐sample *t* test, and data on sex of patients with MMD and healthy controls were presented as *n* (%) and compared using Pearson chi‐square test. The EPC counts and CEC counts were represented as a mean with standard error (*SE*) of patients with MMD and healthy controls, and both outcomes were compared between the two groups using two‐sample *t* test. A Pearson correlation analysis was applied to identify the correlation between EPC counts and CEC counts in patients with MMD. The results were presented as a coefficient of correlation with corresponding *p*‐value. Univariate linear regression was used to identify the association of either EPC counts or CEC counts with the clinical characteristics in patients with MMD. Variables with significant association (*p* < 0.05) were selected and put into subsequent multiple linear regression analysis. Results were presented as the estimated *β* value with standard error (*SE*) and *p*‐value. With regard to statistical power, the study was planned to have 65 healthy controls and 81 patients with MMD. The statistical power was derived as 72.9% and 92.0% to evaluate the means of EPCs/PBMCs and the difference of CECs and PBMCs between healthy controls and patients with MMD based on Type I error probability = 0.05. All statistical assessments were two‐tailed and considered significant at *p* < 0.05. All statistical analyses were carried out with IBM SPSS statistical software version 22 for Windows (IBM Corp., Armonk, NY, USA).

## 3 RESULTS

### 3.1 Patient population

This study enrolled 66 patients with MMD (29 males/37 females) and 81 healthy controls (36 males/45 females). There was no significant difference in age or sex between the patients with MMD and healthy controls (Table 1).

**Table 1:** Demographics of patients with MMD and healthy controls

Demographics	Number of patients (*n* = 66)	Number of healthy controls (*n* = 81)	*p*‐value
Age	41.4 ± 10.4 (range: 23 to 71)	39.1 ± 10.9 (range: 19 to 65)	0.205
Sex			
Female	37 (56.1)	45 (55.6)	1.000
Male	29 (43.9)	36 (44.4)	

*Notes*

Data are presented as means ± standard deviations (*SD*) with a range (min. to max.) for age and *n* (%) for sex.

MMD: moyamoya disease.

All of the relevant clinical characteristics of the patients with MMD are summarized in Table 2. The initial symptom in 36 patients (54.5%) was transient ischemic attack (TIA) and in 28 (42.5%) infarction, and two patients (3.0%) were asymptomatic. Thirty‐eight patients were diagnosed with either hypertension, diabetes mellitus (DM), or coronary heart disease. According to the Suzuki Angiographic Stage, 21 patients were in stage 6, 16 in stage 5, 16 in stage 4, and 13 in stage 3. In the serological examination, 14 patients were diagnosed with hyperhomocysteinemia, 16 with hypertriglyceridemia, and 1 with hypercholesterolemia. Among all patients, 21 had no moyamoya vessels, 16 had a small amount of moyamoya vessels, and 29 had a large amount of moyamoya vessels.

**Table 2:** Clinical characteristics of patients with MMD

Patient characteristics	Number of patients (*n* = 66)	Range
Family history	3 (4.5)	
Clinical history	38 (57.6)	
Hypertension	24	
Diabetes mellitus	6	
Coronary heart disease	3	
Hypertension & diabetes mellitus	3	
Hypertension & coronary heart disease	2	
Initial symptoms		
TIA	36 (54.5)	
Infarction	28 (42.5)	
Asymptomatic	2 (3.0)	
Side of lesions		
Single	10 (15.2)	
Bilateral	56 (84.8)	
PCA involvement	24 (36.4)	
Concomitant disease	27 (40.9)	
Suzuki Angiographic Stage		
1	0 (0)	
2	0 (0)	
3	13 (19.7)	
4	16 (24.2)	
5	16 (24.2)	
6	21 (31.9)	
Serological examination		
Homocysteine, μmol/L	13.94 ± 8.14	Range: 4.8 to 55
Hyperhomocysteinemia	14 (21.2)	
CRP, mg/L	4.01 ± 8.29	Range: 0.3 to 33
Apolipoprotein A1, g/L	1.16 ± 0.22	Range: 0.72 to 1.76
Apolipoprotein B, g/L	0.78 ± 0.22	Range: 0.31 to 1.39
Total cholesterol, mmol/L	4.04 ± 1.03	Range: 2.00 to 6.49
HDL‐c, mmol/L	1.13 ± 0.24	Range: 0.63 to 1.66
LDL‐c, mmol/L	2.13 ± 0.72	Range: 0.67 to 3.70
Hypercholesterolemia	1 (1.5)	
Triglyceride, mmol/L	1.43 ± 0.77	Range: 0.38 to 3.97
Hypertriglyceridemia	16 (24.2)	
Amount of Moyamoya vessels		
None (Suzuki angiographic stage 1,6)	21 (31.8)	
Small (Suzuki angiographic stage 2,5)	16 (24.2)	
Large (Suzuki angiographic stage 3,4)	29 (43.9)	

*Notes*

Data are presented as *n* (%) for categorical variables and means ± standard deviations (*SD*) with a range (min. to max.) for continuous variables.

Patients were identified as having homocysteinemia if homocysteine >15 μmol/L; hypercholesterolemia if total cholesterol >6.2 mmol/L; and hypertriglyceridemia if triglyceride >1.81 mmol/L.

### 3.2 Quantification of CECs and EPCs

**Figure 1:**
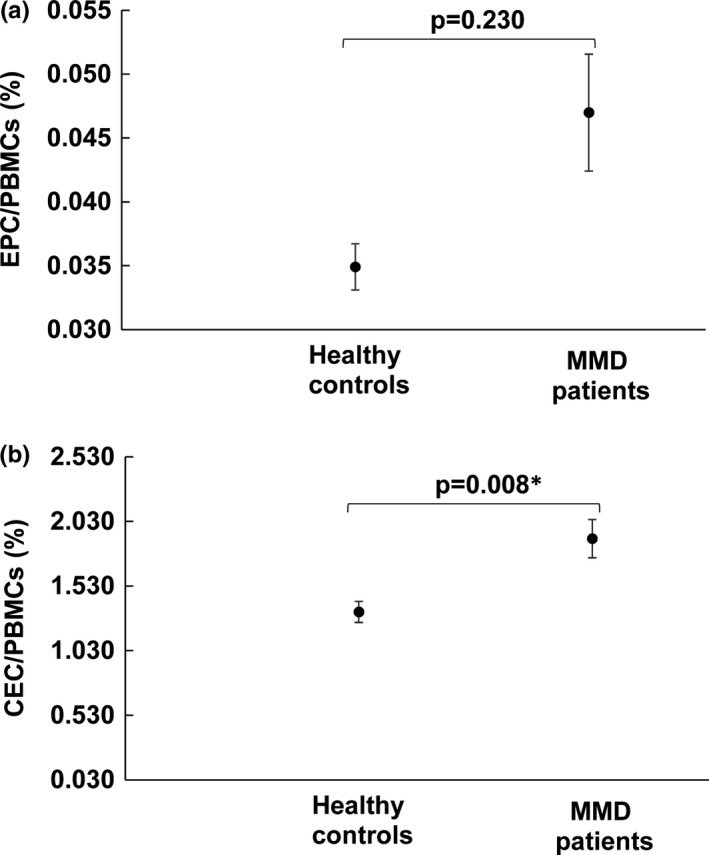
Comparison of EPC counts (a) and CEC counts (b) between patients with MMD and healthy controls. Data are presented as a mean ± *SE*. *p < 0.05 indicates significant difference between two groups

Endothelial progenitor cell counts in the patients with MMD were higher (0.046% ± 0.037% of blood mononuclear cells) than in the healthy controls (0.035% ± 0.016% of blood mononuclear cells). However, it did not reach statistical significance (*p* = 0.230) (Figure 1a). The CEC counts in the patients with MMD were significantly higher (1.897% ± 1.203% of blood mononuclear cells) than in the healthy controls (1.330% ± 0.735% of blood mononuclear cells) (*p* = 0.008) (Figure 1b). Pearson correlation analysis showed that CEC counts were positively correlated with EPC counts. However, it did not reach statistical significance (coefficient of correlation *r* = 0.025; *p* = 0.762) (results not shown).

Table 2 shows the results of univariate linear regression of EPC and CEC counts with regard to the patients’ clinical characteristics. EPC counts were associated with age (*p* = 0.020), and the concomitant disease (e.g., hypertension/diabetes and coronary heart disease) was also considered into following multivariate analysis of EPC counts although the significance was at borderline (*p* = 0.054). However, in multivariate analysis EPC counts were only associated with the age of patients with MMD without including concomitant disease (age: *β* = −0.009, *p* = 0.049) (Supporting Information Table S1).

In the univariate analysis in Table 3, the two variables that were significantly associated with EPC counts, concomitant disease and age, were retained and put into subsequent multivariate analysis. CEC counts were only significantly associated with concomitant disease (*β* = 0.649, *p* = 0.030). And the moyamoya vessels were also considered in the following multivariate analysis of CEC counts although the significance for a large amount of moyamoya vessels was borderline (*β* = 0.622, *p* = 0.073). The results showed a significant association between concomitant disease and CEC counts (*β* = 0.564, *p* = 0.039) (Supporting Information Table S1).

**Table 3:** Univariate linear regression of EPC counts and CEC counts with regard to all the characteristics of patients with MMD (*N* = 66)

Variables	Association with EPC/PBMCs		Association with CEC/PBMCs	
	Β (*SE*)	*p*‐value	Β (*SE*)	*p*‐value
Age, years	−0.001 (0.0004)	**0.020** a	−0.020 (0.014)	0.164
Sex, females vs. males	−0.004 (0.009)	0.675	−0.004 (0.009)	0.675
Having family history	−0.025 (0.022)	0.255	−0.914 (0.707)	0.201
Initial symptoms				
TIA	0.027 (0.026)	0.311	0.027 (0.026)	0.311
Infarction	0.009 (0.027)	0.749		
Asymptomatic	0		0	
Side of lesions				
Single	0		0	
Bilateral	−0.004 (0.013)	0.753	0.392 (0.413)	0.346
PCA involvement	−0.009 (0.010)	0.363	0.033 (0.310)	0.916
Concomitant disease	−0.018 (0.009)	**0.054**	0.649 (0.293)	**0.030** a
Suzuki Angiographic Stage				
1	ND	NA	ND	NA
2	ND	NA	ND	NA
3	0.015 (0.013)	0.254	0.015 (0.013)	0.254
4	0.0003 (0.012)	0.980	0.0003 (0.012)	0.980
5	0.012 (0.012)	0.339	0.012 (0.012)	0.339
6	0		0	
Serological examination				
Homocysteine, μmol/L	−0.0002 (0.0010)	0.757	0.006 (0.020)	0.774
CRP, unit	−0.0003 (0.0008)	0.658	0.004 (0.027)	0.881
Apolipoprotein A1, g/L	0.033 (0.022)	0.131	0.681 (0.685)	0.324
Apolipoprotein B, g/L	0.009 (0.022)	0.687	−0.986 (0.679)	0.152
Total cholesterol (mmol/L)	0.006 (0.005)	0.229	−0.087 (0.144)	0.547
HDL‐c, mmol/L	0.025 (0.019)	0.192	0.570 (0.596)	0.343
LDL‐c (mmol/L)	0.007 (0.006)	0.284	−0.181 (0.205)	0.381
Triglyceride (mmol/L)	0.005 (0.006)	0.396	−0.359 (0.189)	0.062
Amount of moyamoya vessels				
None	0		0	
Small	0.012 (0.012)	0.339	0.503 (0.395)	0.207
Large	0.007 (0.011)	0.517	0.622 (0.341)	0.073

*Notes*

Results were presented as the estimated *β* with corresponding standard error (*SE*) and *p*‐value.

CEC: circulating endothelial cells; EPC: endothelial progenitor cells; ND: not derived; NA: not assessed; PCA: posterior cerebral artery.

a Numbers in bold indicates significant association (*p* < 0.05).

The mean modified Rankin Scale (mRS) score was 1.03 (*SD* = 1.34). Thirty‐four patients had a score of 0, 13 a score of 1, 8 a score of 2, 5 a score of 3, and 6 a score of 4. The results of correlation analysis showed mRS score was negatively correlated with EPC counts (*r* = −0.285, *p* = 0.022). However, there was no significant correlation between CEC counts and mRS score (*r* = −0.048, *p* = 0.703).

## 4 DISCUSSION

Our study is the first to investigate the presence of CECs in the plasma of patients with MMD. CECs have been used as a marker in a variety of vascular disorders, and our results of increased CECs in MMD provide evidence that vascular injury is crucial for the development of MMD. Our most important finding was that the amount of CECs was negatively correlated with concomitant disease such as hypertension, diabetes mellitus, and coronary heart disease. We also found that patients with MMD had increased EPC and CEC counts and that EPC counts were independently associated with patient age. Our results might suggest further avenues of research to understand the pathogenesis of the condition.

Research in the past has focused on measuring the levels of inflammatory molecules, cytokines, chemokines, and growth factors in serum and CSF in patients with MMD (Bedini et al., 2016). More recently, investigators have begun to use the number and function of circulating cells to gain insights into the pathogenesis of MMD. The focus has been on bone marrow‐derived progenitor cells playing a role in repairing the endothelium as well as circulating endothelial cells (CECs) as markers of endothelial injury or activation. A number of studies have investigated abnormal EPC and CEC number and function in various disease states; however, lack of uniformity in terminology and methodology has caused considerable confusion in characterizing these cells. Flow cytometry has the advantage of being sensitive, reproducible, and relatively easy to perform and is the method of choice to directly detect circulating progenitor cells. However, there are limitations—the relative rarity of these cells and the difficulty in characterizing them simply on the basis of surface antigens. Several authors have used polychromatic flow cytometry which can even better characterize these circulating cells (Duda et al., 2007). Using this protocol, we found that two populations were detectable in numbers (Figure 1). One population consisted of CD31^bright^CD34^dim^CD45^−^CD133^−^ cells (referred to as CECs), and the other was represented by CD31^+^CD34^bright^CD133^+^CD45^dim^ (referred to as EPCs).

This initial study is the first to assess CEC levels in MMD, and the results suggest that CEC levels are significantly higher in patients with MMD as compared with healthy controls. Endothelial cells (ECs) can detach from the vascular wall and circulate in the bloodstream, and would then be termed CECs. It has long been shown that the level of CECs is significantly higher in patients with widespread vascular damage, such as antineutrophil cytoplasmic antibody‐associated small vessel vasculitis (Woywodt, Streiber, et al., 2003), sickle cell crisis (Belcher et al., 2003), and pulmonary hypertension (Bull et al., 2003). Therefore, CECs have been used as a marker of endothelial damage in a variety of vascular disorders (Alessio et al., 2013; Davignon & Ganz, 2004; Dignat‐George & Sampol, 2000). Elevated CEC levels have been observed in various pathological conditions associated with vascular disease and are considered by some to be a biomarker of disease severity in vascular conditions (Erdbruegger, Haubitz, & Woywodt, 2006). Indeed, the vascular endothelium is intimately linked with a variety of conditions including cardiovascular, autoimmune, infectious, and neoplastic diseases (Bertolini, Shaked, Mancuso, & Kerbel, 2006; Bull et al., 2003; Clancy et al., 2001; Goon, Boos, & Lip, 2005; Percivalle, Revello, Vago, Morini, & Gerna, 1993; Quilici et al., 2004; Woywodt, Schroeder, et al., 2003). Vascular injury represents a major initiating step in the pathogenesis of atherosclerosis. Vascular injury is followed by lipid accumulation, monocyte and platelet adhesion, and smooth muscle cell proliferation resulting in plaque formation (Willerson, 2002). Similarly, MMD has been characterized by prominent vascular changes probably caused by endothelium injury, followed by intima thickening composed of fibrocellular materials, and smooth muscle cells proliferating (Fukui, Kono, Sueishi, & Ikezaki, 2000). Thus, CECs may serve as an in vivo indicator of vascular injuries and may enable probing into the functional and metabolic changes of in vivo endothelial cells in different vascular diseases. We postulate that endothelial cell injury is the inciting event in MMD pathology under some conditions, such as infections, immunological defects, or hemodynamic stress, particularly in individuals with a specific genetic background and/or angiogenic abnormality.

In this study, we found a significant relationship between EPC counts and age. In a study of patients with type 1 diabetes, it was found that younger patients (age < 20 years) had significantly higher circulating EPC counts than adult patients (Arcangeli et al., 2017). Disease duration has no effect on this finding. This suggests that younger patients may have greater protection against vascular damage than older patients.

Previous studies reported that CEC counts only reflected endothelium injury, but did not correlate with age, gender, serum cholesterol, hypertension, obesity, history of cardiovascular disease, glucose levels, or smoking (Clancy et al., 2001; Goon et al., 2005). These results are in accordance with our study. As vascular injury exists in hypertension, diabetes mellitus, and coronary heart disease, MMD concomitant with these diseases may lead to superimposed damage to the endothelium. That could explain our results. The results of a study by Wihastuti, Sargowo, Agoes, and Satuman Heriansyah (2014) might be of value in obtaining further understanding of our findings. They studied three groups: a healthy group, a group with vascular risk factors, and a group with vascular disease (coronary heart disease, diabetes, or stroke). They found that the group with vascular disease had the highest levels of CECs and the healthy group the lowest levels. They concluded that CEC expression is related to both vascular disease and risk factors for vascular disease. CECs can be derived from the vascular wall (mature CECs), or EPCs (Asahara et al., 1997). Our results showed that CEC counts had no significant correlation with EPC counts (*p *> 0.05). From the results, we deduced that CECs may be derived from the vascular wall.

It should be noted that in this study, high EPC counts correlated with better mRS score. This could be interpreted to mean that good angiogenesis leads to less permanent brain damage. The percentage of patients with posterior cerebral artery (PCA) involvement in our study was 34.5%, which might be considered high. However, similar levels of PCA involvement have been reported in other studies (Kim et al., 2016; Lee, Kim, Phi, & Wang, 2015).

It is possible that increased levels of CECs and EPCs in MMD patients with hypertension and/or coronary heart disease could solely be due to concomitant disease. However, the increased levels could be attributable to concomitant disease and MMD.

There are certain limitations to this study that should be noted. The most important one is the already mentioned difficulty in characterizing CECs and EPCs. It is unreasonable to compare our results with other studies using distinct markers for CEC and EPC evaluation in patients without a standardization of the protocol. Another limitation is that we did not confirm our results using EPC culture assay, such as CFU‐EC and ECFC. However, our idea was to perform a pilot and exploratory study comparing results obtained in patient with MMD and healthy controls. We hope that our results and the insights obtained into the phenotype of CECs and EPCs provide data to assist further research studies in MMD. In addition, we did not study EPCs and CECs in different stages of the same patient with MMD. However, we have already studied many different stages of MMD (e.g., different blood vessel stages, different clinical type stages). A possible confounding factor was that the cohort was fairly heterogeneous. The patients had different initial symptoms, and 28 patients had concomitant disease. Because the symptoms of MMD are so varied it is difficult to have a cohort of patients with the same symptoms (Kim, 2016).

In conclusion, this study is the first to investigate the presence of CECs in the plasma of patients with MMD. We found a negative correlation between CEC count and concomitant disease such as hypertension, diabetes mellitus, and coronary heart disease. Our results may be useful to further understanding of the pathogenesis of MMD.

## CONFLICT OF INTEREST

None declared.
