# Supplementary material for: Circulating endothelial progenitor cells and endothelial cells in moyamoya disease
Source: Brain Behav. 2018 Aug 23;8(9):e01035. doi: 10.1002/brb3.1035 (PMC6160662; doi:10.1002/brb3.1035)
Supplement: Supplementary file 1 [file BRB3-8-e01035-s001.doc]

**Supplementary Table S1. Multivariate linear regression of EPC counts and CEC counts with regard to significant characteristics of MMD patients in univariate analysis. (N=66)**

|  | Association with EPC / PBMCs | |  | Association with CEC / PBMCs | |
| --- | --- | --- | --- | --- | --- |
| Variables | Β (SE) | p-value |  | Β (SE) | p-value |
| Age, years | -0.0009 (0.0004) | **0.049*** |  | - |  |
| Concomitant disease | -0.014 (0.009) | 0.138 |  | 0.656 (0.303) | **0.034*** |
| Amount of moyamoya vessels |  |  |  |  |  |
| None | - |  |  | 0 |  |
| Small | - |  |  | 0.630 (0.388) | 0.109 |
| Large | - |  |  | 0.510 (0.335) | 0.133 |

Results were presented as the estimated β with corresponding standard error (SE) and p-value. -, not included.

*indicates significant association (p<0.05)
